# First-spike coding promotes accurate and efficient spiking neural networks for discrete events with rich temporal structures

**DOI:** 10.3389/fnins.2023.1266003

**Published:** 2023-10-02

**Authors:** Siying Liu, Vincent C. H. Leung, Pier Luigi Dragotti

**Affiliations:** Communications and Signal Processing Group, Department of Electrical and Electronic Engineering, Imperial College London, London, United Kingdom

**Keywords:** spiking neural networks, first-spike coding, firing rate coding, time-to-first-spike, surrogate gradient, event-based data, temporal structures

## Abstract

Spiking neural networks (SNNs) are well-suited to process asynchronous event-based data. Most of the existing SNNs use rate-coding schemes that focus on firing rate (FR), and so they generally ignore the spike timing in events. On the contrary, methods based on temporal coding, particularly time-to-first-spike (TTFS) coding, can be accurate and efficient but they are difficult to train. Currently, there is limited research on applying TTFS coding to real events, since traditional TTFS-based methods impose one-spike constraint, which is not realistic for event-based data. In this study, we present a novel decision-making strategy based on first-spike (FS) coding that encodes FS timings of the output neurons to investigate the role of the first-spike timing in classifying real-world event sequences with complex temporal structures. To achieve FS coding, we propose a novel surrogate gradient learning method for discrete spike trains. In the forward pass, output spikes are encoded into discrete times to generate FS times. In the backpropagation, we develop an error assignment method that propagates error from FS times to spikes through a Gaussian window, and then supervised learning for spikes is implemented through a surrogate gradient approach. Additional strategies are introduced to facilitate the training of FS timings, such as adding empty sequences and employing different parameters for different layers. We make a comprehensive comparison between FS and FR coding in the experiments. Our results show that FS coding achieves comparable accuracy to FR coding while leading to superior energy efficiency and distinct neuronal dynamics on data sequences with very rich temporal structures. Additionally, a longer time delay in the first spike leads to higher accuracy, indicating important information is encoded in the timing of the first spike.

## 1. Introduction

The emergence of event-driven neuromorphic devices has given further impetus to the development of spiking neural networks (SNNs) (Anumula et al., 2018). SNNs more closely mimic biological neural systems by processing and transmitting information with sparse and asynchronous binary spikes (Pfeiffer and Pfeil, 2018). By incorporating spike timing in their neuron model, SNNs have become effective tools for acquiring and processing temporal information (Wang et al., 2020). Neuromorphic devices such as dynamic vision sensors (DVS) and dynamic audio sensors (DAS) produce asynchronous events which are well-suited to be used as the input of SNNs. Combining SNNs with the output of neuromorphic devices can potentially enable the development of power-efficient systems that more closely mimic biological processing.

SNNs process a sequence of spikes in each layer, which is referred to as spike trains. A spike train is mathematically defined by $s\left(t\right)=\underset{t_{i}\in I}{\sum }\delta \left(t-t_{i}\right)$, where *t*_*i*_ represents the timing of individual spikes in the set I. In terms of information encoding, rate coding and temporal coding are two distinct approaches in SNNs (Rullen and Thorpe, 2001; Huxter et al., 2003; Brette, 2015; Kiselev, 2016; Liu and Wang, 2022). Rate coding focuses on the firing rate (FR) of neurons, in which information is represented by the average number of spikes within a certain time window. Although it is widely used and effective, rate coding is less efficient because it involves a great number of spikes and ignores the relative timing between spikes, which encodes important information of stimulus in visual (Gollisch and Meister, 2008), auditory (Heil, 2004; Fontaine and Peremans, 2009), and other systems (Panzeri et al., 2001; Huxter et al., 2003). Alternatively, temporal coding schemes rely on the precise timing of individual spikes, and this potentially provides a faster and efficient way of processing and transmitting signals. In particular, the first spike after a stimulus (Panzeri et al., 2001; Johansson and Birznieks, 2004) is capable of reliably conveying considerable information. This inspired methods based on the time-to-first-spike (TTFS) coding, resulting in fewer spikes and efficient computation (Bonilla et al., 2022; Yu et al., 2023). In practice, most of these methods force each neuron to fire at most one spike (Mostafa, 2018; Kheradpisheh and Masquelier, 2020; Göltz et al., 2021; Mirsadeghi et al., 2021; Zhou et al., 2021; Comşa et al., 2022) or assume there is a very long refractory period after a spike (Kotariya and Ganguly, 2021) to allow the computation of exact derivatives of postsynaptic spike times with respect to presynaptic times. This means that these networks can only process static inputs (Mostafa, 2018; Zhou et al., 2021; Comşa et al., 2022; Sakemi et al., 2023), such as spikes converted from intensity of each pixel in images, but not a continuous stream of events. Hence, this type of single-spike encoding is not biologically plausible.

**Figure 1:**
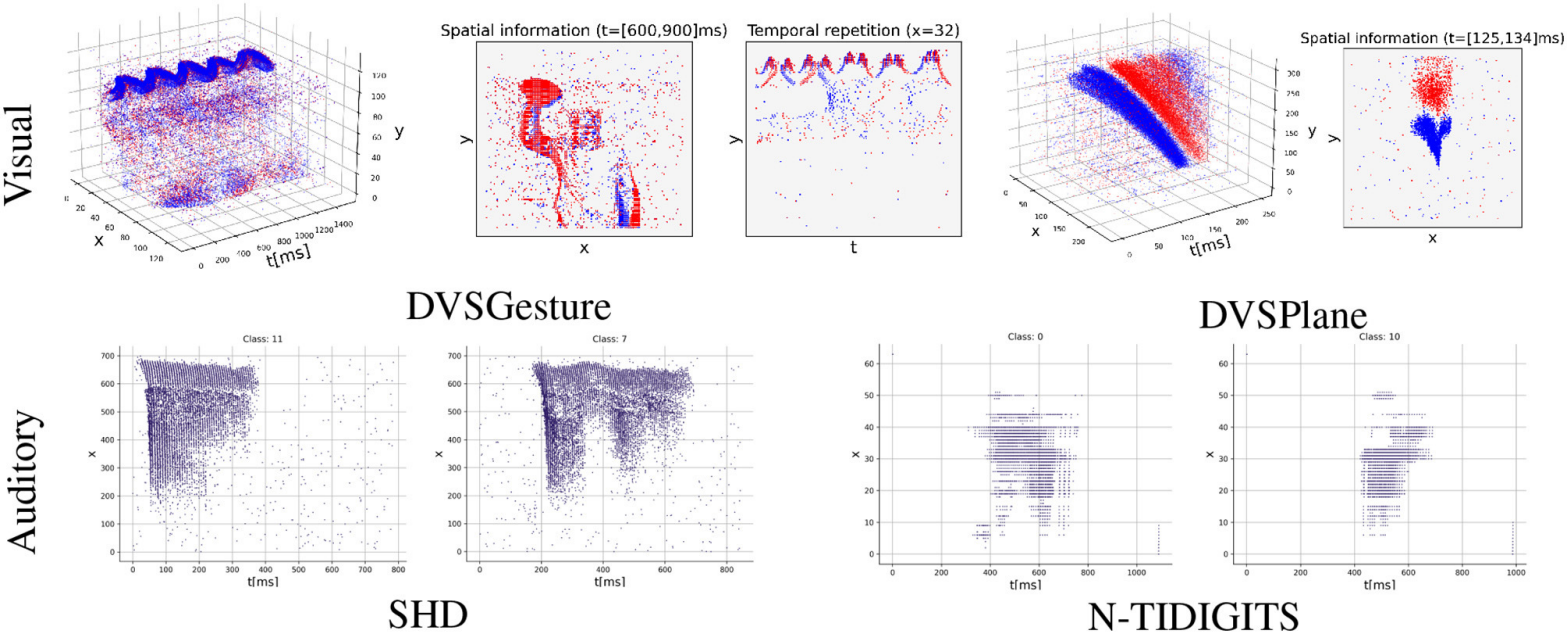
Event sequences with varying degrees of temporal structures (blue/red: positive/negative events). DVSGesture and DVSPlane are visual datasets that heavily rely on the spatial information in decision-making. The sequences of DVSGesture are periodic in the temporal domain, while DVSPlane data exhibits more complex temporal structures as it lacks temporal repetition. On the other hand, audio data sequences in SHD and N-TIDIGITS are non-repetitive and it is difficult to differentiate between classes solely based on spatial information. DVSGesture (Amir et al., 2017), SHD (Cramer et al., 2022), N-TIDIGITS (Anumula et al., 2018), DVSPlane (Afshar et al., 2019).

In addition, there have been limited research on investigating the temporal structures in neuromorphic data sequences and SNNs. In the context of spiking signals, temporal structures refer to the patterns, changes, or behaviors that occur over time in the generation and transmission of these signals. Data sequences containing rich temporal structures indicate that useful information is encoded in the temporal domain. As shown in Figure 1, various types of data produce varying degrees of temporal structures, which can yield diverse results when using different coding schemes. However, some widely used datasets lack diverse temporal structures in their sequences because events are generated by repeatedly moving a neuromorphic device around static images, such as neuromorphic MNIST (N-MNIST), N-Caltech101 (Orchard et al., 2015), and CIFAR10-DVS (Li et al., 2017). Results presented by Iyer et al. (2021) illustrated that rate-based SNNs outperform timing-based methods on N-MNIST dataset. The authors argued that spike timings of sequences in N-MNIST may not contain too much useful information. Moreover, recent evidence (Jiang et al., 2023) indicates that timing-based computation is superior in the task involving abundant temporal information. As for TTFS coding, although some studies focused on event data of static scenes (Park et al., 2020; Kotariya and Ganguly, 2021), there have not been studies applying TTFS coding to real event sequences that exhibit rich temporal structures.

We, therefore, propose a novel decision-making scheme based on first-spike (FS) coding by encoding FS timings of output neurons for real-world event sequences with rich temporal structures, aiming to understand whether the timing of the FS plays a distinct role and whether it exploits the temporal information more effectively than FR. The FS coding differs from traditional TTFS-based studies in that information is conveyed without relying on exact timings, thereby eliminating the restriction of allowing each neuron to fire at most once. Instead, we only encode the timings of the fist spike in the output layer and use it in the loss function for supervised learning. However, supervised learning involving precise timing poses several challenges. First, it is impractical to use continuous time encoding due to high computational cost caused by the substantial number of events generated by neuromorphic device. In addition, exact gradients of continuous spike times with respect to spike trains are ill-defined, which prevents the standard backpropagation process used in conventional artificial neural networks (ANNs). Some existing methods overcome this issue by estimating the derivatives of continuous time with respect to membrane potential around the threshold, such as SpikeProp (Bohte et al., 2002) and its variations (Xu et al., 2013; Shrestha and Song, 2016, 2017) and EventProp (Wunderlich and Pehle, 2021). Other methods utilize the relationship between time and membrane potential to achieve supervised learning of precise spike timing, such as probabilistic models of firing intensity (Pfister et al., 2006; Gardner et al., 2015) and implicit differentiation on the equilibrium state (Xiao et al., 2020, 2023). Recent methods have made attempts to calculate exact derivatives of the postsynaptic spike times with respect to presynaptic spike times (Comşa et al., 2022) or potential (Zhang et al., 2022). For example, Zhang et al. (2022) proposed a rectified linear postsynaptic potential function to alleviate problems such as non-differentiable spike function, exploding gradients and dead neurons during backpropagation in deep SNNs utilizing temporal coding. Most methods train the network to learn the timing of desired spike trains, restricting its adaptability to diverse input scenarios. Furthermore, the complicated rules of error propagation and the dependency between spike times in these methods limit their utilization in deep networks. Therefore, to simplify training and alleviate the restrictions for spike times, we exclusively apply discrete temporal coding to the output spikes in the final layer. In this way, we can concentrate on error propagation from output FS timings to subsequent spikes by leveraging the surrogate gradient learning (Wu et al., 2018; Neftci et al., 2019; Yin et al., 2021) for spikes. Specifically, the error of FS time in the output layer is propagated to multiple spikes through a Gaussian window, and then the SuperSpike method (Zenke and Ganguli, 2018), based on surrogate gradient descent, is utilized to achieve the supervised learning of spikes in the network. Additionally, this approach enables a flexible configuration of the network architecture, which can include a combination of convolutional layers and fully connected (FC) structures with recurrent connections.

Another difficulty in training SNNs is dealing with neurons that fail to generate any spikes within the given time window, commonly referred to as inactive neurons. This issue is particularly predominant in training based on the first-spike time, as the error is only propagated through the first output spike. Consequently, the weights associated with subsequent spikes cannot be updated, leading to a lower firing rate and increasing inactivity in their neurons. Additional strategies are usually necessary to solve this problem, such as weight initialization (Bohte et al., 2002), large penalty on inactive neurons (Mostafa, 2018; Comşa et al., 2022), and synchronization pulses as temporal biases (Comşa et al., 2022). Hence, in this study, we design strategies to facilitate the training based on FS timings, specifically for event sequences. First, we assign the error of inactive neurons across multiple steps rather than just one. Second, the time window is enlarged by adding empty sequences to reduce the number of inactive neurons that generate spikes beyond the observed window due to significant time delay. Finally, to enhance the performance of FS coding, we apply smaller values of time constant and threshold in the initial layers to effectively extract local features, while we use large values in the final layers to facilitate decision-making based on previous stimuli.

In the experiments, we make a comprehensive comparison of FS and FR coding schemes on several commonly used visual and auditory neuromorphic datasets, including DVSGesture (Amir et al., 2017), SHD (Cramer et al., 2022), N-TIDIGITS (Anumula et al., 2018), and DVSPlane (Afshar et al., 2019). These data sequences demonstrate different levels of temporal structures, as shown in Figure 1. Results show that FS coding achieves comparable accuracy with FR coding, although typically with a lower temporal delay. There is a trade-off between classification accuracy and the first-spike latency. An appropriate temporal delay allows the network to make accurate decisions after receiving sufficient information. Furthermore, the FS models exhibit distinct neuronal behavior on different types of data sequences. In particular, the networks based on FS coding demonstrate enhanced performance and superior energy efficiency on audio data sequences with very rich temporal structures. On the other hand, when processing visual data sequences containing repetitive signals and rich spatial information, FS and FR models demonstrate similar neuronal dynamics and produce similar spike counts.

## 2. Materials and methods

Consider a stream of events emitted by a neuromorphic sensor, $\varepsilon =\left\{e_{i},i=1,2,\cdots \;,M\right\}$, over a certain time window. An event *e*_*i*_ in continuous space and time can be represented as a function *e*_*i*_(***x***, *t*) = *p*_*i*_δ(***x***−***x***_*i*_, *t*−*t*_*i*_), which means that an event with polarity *p*_*i*_ is emitted at the location ***x***_*i*_ and at the timestamp *t*_*i*_. The polarity *p*_*i*_ = ±1 represents whether the brightness change is positive or negative. To reduce computational cost, we transform ε into a discretized spatio-temporal representation ***E*** as the input of SNNs. The input tensor contains *T* temporal bins by accumulating raw events at a resolution of Δ*t*. Each pixel location takes the number of positive or negative events within each temporal bin. In this setup, every pixel is associated with two channels to indicate the polarity of events. As a result, for a vision sensor with an image plane of dimensions *H*×*W*, the input ***E*** forms a 4-D tensor of size 2 × *H*×*W*×*T*. As audio sensors have no polarity applied, the input ***E*** is represented as a 2-D tensor with dimensions *F*×*T*, where *F* denotes the number of channels for the audio sensor.

**Figure 2:**
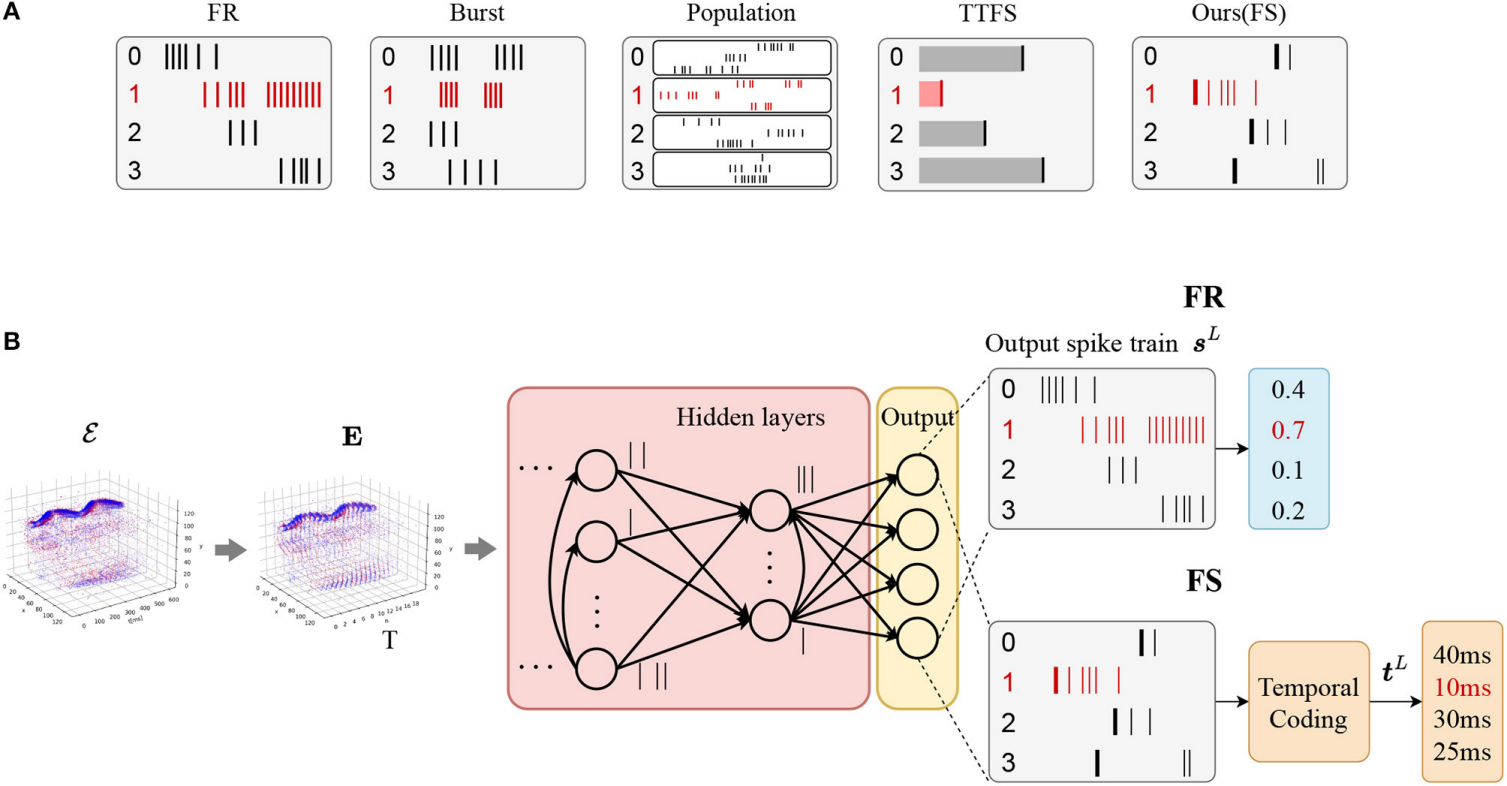
Coding schemes and the SNN architecture. **(A)** Comparisons of different spike-based coding in decision-making. Our proposed FS coding is only applied to the output layer. **(B)** The SNN architecture for the classification problem using FR **(top)** and FS **(bottom)** coding for the output layer. The input events are represented with spatio-temporal grids ***E***. The output spike trains in the final layer are encoded into FR and FS timings for classification. For FS coding, the predicted class corresponds to the neuron which fires the earliest spike, while for FR coding, the predicted class corresponds to the neuron which fires more spikes.

In terms of the coding schemes of SNNs, spike trains can be encoded into different formats to convey information (Guo et al., 2021). Figure 2A presents the comparison of spike-based coding schemes in decision-making. FR coding focuses on the average spike count within a certain time window. In population coding, several neurons in each population capture different features of input stimuli over time, and their responses are combined to make a decision (Panzeri et al., 2015). In burst coding, a burst of spikes is emitted at one time, in which information is carried in the spike count and the inter-spike interval within the burst (Izhikevich et al., 2003). Traditional TTFS coding restricts each neuron to fire at most once and information is conveyed in the exact timings, while our FS coding only focuses on the first spike of output neurons, since, in FS, the output neuron that fires first determines the classification outcome.

A standard multi-layer SNN architecture is used in this study, and is implemented using either convolutional layers or fully connected layers based on the required task. The overall dynamics is shown in Figure 2B. It takes the spatio-temporal representation of events ***E*** as the input, and each neuron generates a spike train of length *T*. In the output layer, FR or FS coding are used for classification. The models using FR or FS coding are denoted as the FR or FS model in the rest of the article. For the FR model, the predicted class is determined by the highest firing rate. For the FS model, output spike trains are encoded into temporal codes to obtain FS timing for each neuron. The predicted class in this case depends on the earliest spike across all the output neurons.

Our SNN model can be seen as a hybrid system in which multiple spikes are transmitted in hidden layers, but only the first output spike is utilized to make a decision. However, the information within the hidden layers not only depends on the firing rate (FR) of neurons but also considers the order in which the spikes occur. This is because the first-spike timing is emphasized in the output layer, introducing the aspect of spike order as an informative factor. This distinguishes it from standard FR coding, where only the spike count over a certain time window holds significance.

We introduce the SNN model with discrete time encoding in Section 2.1, the error propagation through FS timings in Section 2.2, and strategies facilitating the training based on FS timings in Section 2.3.

### 2.1. SNN model and time encoding

In this subsection, we introduce the current-based leaky integrate-and-fire (CUBA-LIF) neuron model in Section 2.1.1 and then extend it to a multi-layer SNN for event sequences in Section 2.1.2. In our SNN model, binary spikes are transmitted and processed between layers, and discrete time encoding is applied to the spike trains of the output layer to obtain the FS timings of the system.

#### 2.1.1. Neuron model

One of the most commonly used neuron model is the CUBA-LIF neuron. Consider a set of presynaptic neurons *j* = 1, 2, ⋯ , *J* connected to a postsynaptic neuron *i*, then the dynamics of the CUBA-LIF model is as follows:


(1) $$\begin{array}{l} \begin{array}{cc} {\;\tau }_{s}\frac{dI_{i}\left(t\right)}{dt} & =-I_{i}\left(t\right)+\underset{j}{\sum }w_{ij}\underset{t_{j,m}<t}{\sum }\delta \left(t-t_{j,m}\right),\\ \tau_{m}\frac{dU_{i}\left(t\right)}{dt} & =-\left(U_{i}\left(t\right)-U_{r}\right)+I_{i}\left(t\right), \end{array} \end{array}$$

where τ_*m*_ and τ_*s*_ are the time constant of membrane potential *U*(*t*) and synaptic current *I*(*t*), respectively, and δ(*t*) is the Dirac function representing a spike function. Here, *t*_*j, m*_<*t* is the firing time of the *m*th spike generated by the *j*th presynaptic neuron. Moreover, the synaptic weight between neurons *i* and *j* is denoted as *w*_*ij*_, and *U*_*r*_ is the resting potential, where we set *U*_*r*_ = 0.

The condition that neuron *i* fires a spike is when the membrane potential *U*(*t*) reaches a threshold θ. After spiking, the potential drops below *U*_*r*_ and then recovers to *U*_*r*_ within a refractory period. In our model, the refractory period is ignored, which means *U*_*i*_(*t*) is reset to *U*_*r*_ = 0 instantly.

#### 2.1.2. SNN model with discrete time encoding

The CUBA-LIF neuron model is then discretized to construct a multi-layer SNN. Given an SNN model with *L* layers, the membrane potential ***U***^*l, n*^ is evolved through layers *l* = 1, 2, ⋯ , *L* and time steps *n* = 1, 2, ⋯ , *T*. When the membrane potential of neuron *i* in layer *l* at time step *n* is greater than a threshold: $U_{i}^{l,n}\geq \theta$, a spike is generated and is denoted as $s_{i}^{l,n}=1$. Otherwise $s_{i}^{l,n}=0$.

We follow the same discretization scheme used by Neftci et al. (2019). The update of synaptic current $I_{i}^{l,n}$, membrane potential $U_{i}^{l,n}$, and spike firing from step *n* to *n*+1 are as follows:


(2) $$\begin{array}{ll} I_{i}^{l,n+1} & =\beta I_{i}^{l,n}+\overset{Q\left(l-1\right)}{\underset{j=1}{\sum }}w_{ij}s_{j}^{l-1,n+1}+\overset{Q\left(l\right)}{\underset{k=1}{\sum }}v_{ik}s_{k}^{l,n+1}, \end{array}$$

(3) $$\begin{array}{ll} U_{i}^{l,n+1} & =\alpha U_{i}^{l,n}\left(1-s_{i}^{l,n}\right)+\left(1-\alpha \right)I_{i}^{l,n}, \end{array}$$

(4) $$\begin{array}{ll} s_{i}^{l,n+1} & =f\left(U_{i}^{l,n+1}\right), \end{array}$$

where $\alpha =e^{-\frac{\Delta t}{\tau_{m}}}$ and $\beta =e^{-\frac{\Delta t}{\tau_{s}}}$. Moreover, in our models, τ = τ_*s*_ = τ_*m*_. The last term in Eq. (2) represents optional recurrent connections in the fully connected layer, where *v*_*ik*_ is the weight of a recurrent connection between the *k*th and *i*th neuron in the same layer *l*. The number of neurons in the *l*th layer is denoted as *Q*(*l*). In Eq. (3), the membrane potential $U_{i}^{l,n}$ is reset by multiplying $1-s_{i}^{l,n}$. Finally, the spiking process can be described as a step function of the membrane potential:


(5) $$s=f(U)=\{\begin{array}{cc} 1, & U\geq \theta ,\\ 0, & U<\theta . \end{array}$$

**Figure 3:**
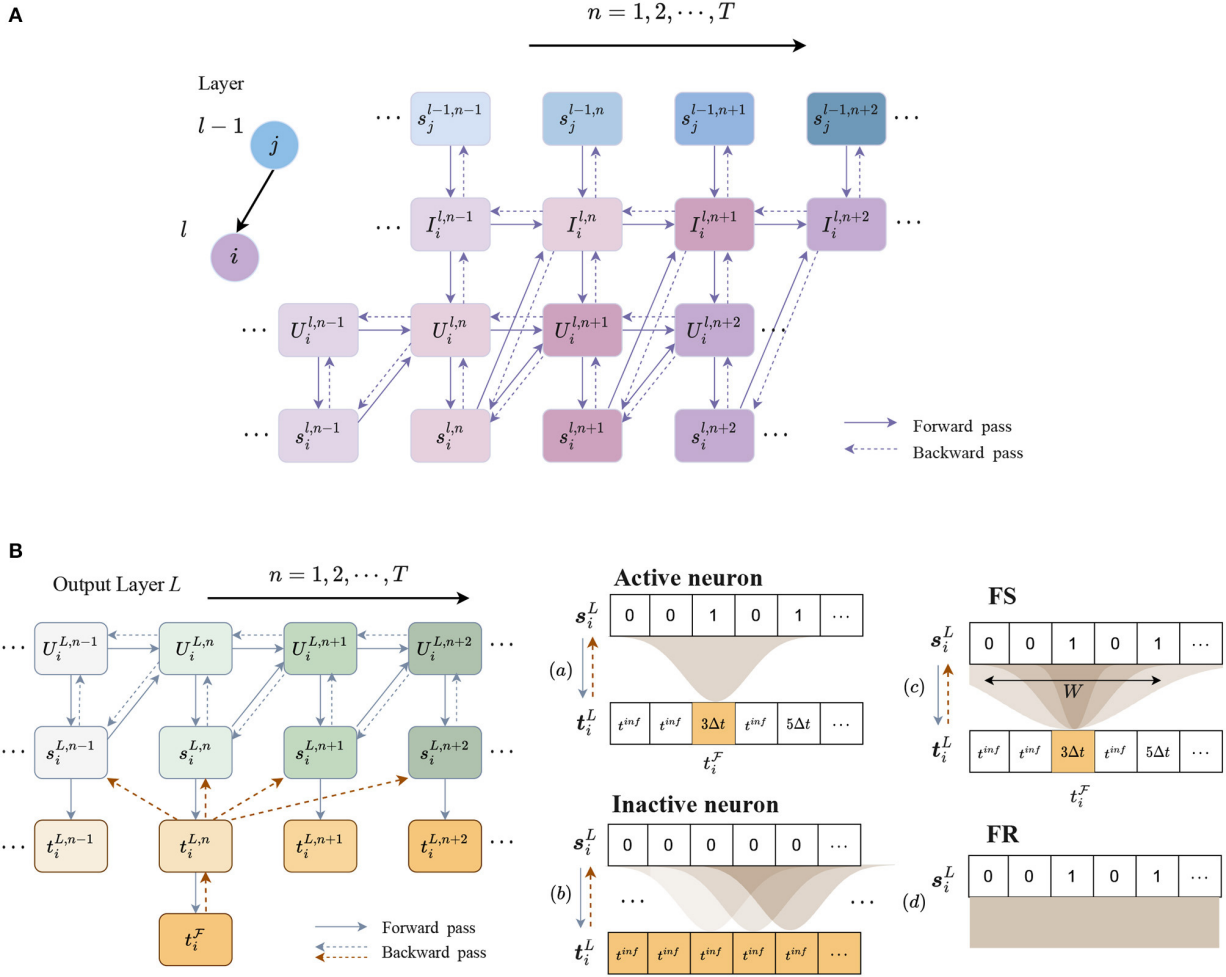
Forward and backward propagation in hidden and output layers. **(A)** Forward and backward propagation of a single neuron through spikes in the SNN model. The *j*th neuron in layer *l*−1 is connected to the *i*th neuron in layer *l*. Connections between $s_{i}^{l}$ and $I_{i}^{l}$ represent recurrent connections. **(B)** iscrete temporal encoding and error assignment from time-to-spike of neuron *i* in the output layer *L*. Output spike train is encoded into discrete times according to the time sequence, while the time of a silent step is encoded as a large value, denoted as $t_{i}^{inf}$. In the backpropagation, a Gaussian filter is used to distribute error from one step to the others. (a) For a valid time of FS, the error $\frac{\partial L}{\partial t_{i}^{F}}$ is propagated to spikes $s_{i}^{L,n}$ through a Gaussian window; (b) for an inactive output neuron, the error is assigned to every time step. (c) As the Gaussian window size *W* increases, the error distribution becomes more similar to the error generated by (d) FR, where each spike is assigned the same error.

The forward propagation of a single neuron is shown as solid arrows in Figure 3A. The synaptic current, membrane potential, and spikes are updated in both spatial and temporal domains using Eqs (3), (4).

The output spike trains of the network can be encoded into different formats for classification. To obtain FS timings of neurons, temporal coding is required to obtain the spike timings. We, therefore, apply discrete time encoding for spike trains of neurons in the last layer *L*. According to the time sequences, a spike is encoded as its time step, but it is unclear how to encode a silent step that does not generate a spike. Directly encoding it as infinity will cause an error in the computation of the loss. We instead replace it with a fixed large value, denoted as *t*^*inf*^, which should be greater than *TΔt*.

Specifically, the output time of the *i*th neuron at step *n* is given by:


(6) $$t_{i}^{L,n}=h(s_{i}^{L,n})=\{\begin{array}{cc} n\Delta t, & s_{i}^{L,n}=1,\\ t^{inf}, & s_{i}^{L,n}=0, \end{array}$$

where


(7) $$\begin{array}{l} t^{inf}=\left(T+1\right)\Delta t. \end{array}$$

The discrete temporal coding process in the last layer *L* is illustrated in Figure 3B. Spikes are encoded into discrete timestamps in accordance with the time sequence, while other steps are encoded with *t*^*inf*^.

Finally, the time of the first spike from the *i*th output neuron, denoted as $t_{i}^{F}$, is given by the minimum of the temporal codes:


(8) $$\begin{array}{l} t_{i}^{F}=\underset{n}{\min}\left(t_{i}^{L,n}\right). \end{array}$$

However, another concern arises when all the neurons fail to fire within the time window. The network cannot make a prediction by the first spike, because it becomes challenging to determine which neuron fires the first. To solve this problem, we utilize the maximum membrane potential over time of each neuron $U_{i}^{max}=\underset{n}{\max}\left(U_{i}^{L,n}\right)$ to facilitate the prediction. The higher the membrane potential, the higher the likelihood the neuron would fire earlier. Therefore, the predicted label *y*^*P*^ corresponds to either the neuron that fires the first spike or the one with the highest membrane potential if all the output neurons are inactive.

### 2.2. Backpropagation through FS timings

In this section, we propose a supervised learning framework for FS coding. First, we define the loss function as the cross-entropy loss based on the FS times of the output neurons. We use this to minimize the FS time of the target neuron and maximize that of non-target neurons:


(9) $$\begin{array}{l} {ℒ}_{FS}=-\sum_{i=1}^{C}y_{i}\log\frac{exp(-\alpha_{0}t_{i}^{ℱ})}{\sum_{j=1}^{C}exp(-\alpha_{0}t_{j}^{ℱ})}\\ \;+\lambda_{t}\sum_{\left\{i|t_{i}^{ℱ}>T\Delta t\right\}}y_{i}[exp(\beta_{0}t_{i}^{ℱ})-1], \end{array}$$

where *y*_*i*_ is the target label (0 or 1) of the *i*th class, *C* is the number of output neurons (classes), and α_0_, β_0_ and λ_*t*_ are constant coefficients, where α_0_ is used to control the speed of training and prevent the exponential function from taking an excessively high value. The second term is a constraint to penalize a target neuron which never fires. For ease of notation, we use L to represent $L_{FS}$ in the rest of the article.

To compare FS with FR coding, similarly, a cross-entropy loss function maximizing the FR of the target neuron is used and is given by:


(10) $${ℒ}_{FR}=-\sum_{i=1}^{C}y_{i}\log\frac{exp(\alpha_{1}f_{i})}{\sum_{j=1}^{C}exp(\alpha_{1}f_{j})},$$

where α_1_ is a constant value and $f_{i}=\frac{1}{T}\overset{T}{\underset{n=1}{\sum }}s_{i}^{L,n}$ is the spike rate within *T* steps of the *i*th neuron in the last layer *L*. Since the training of FR usually does not suffer from inactive neurons, the constraint used in Eq. (9) is not required here.

To learn the weights $W=\left\{w_{ij}^{l}\right\}$ and $V=\left\{v_{ik}^{l}\right\}$, since the discretization described in Eqs (3), (4) effectively leads to an SNN as visualized in Figure 3A, we can simply perform a standard error backpropagation same as in conventional ANNs. However, there remains two challenges for the learning process based on FS timings. First, we have to propagate the error from the FS time $t_{i}^{F}$ to the postsynaptic spikes $s_{i}^{L,n}$ at the output layer. We, therefore, introduce a novel error assignment scheme in Section 2.2.1. Another obstacle is the non-differentiability of the spike function in Eq. (4), which describes the relationship between membrane potential and postsynaptic spikes. This can be overcome by using a surrogate gradient to approximate that of the step function (see Section 2.2.4).

#### 2.2.1. Error propagation from FS times to spikes

The error of the FS time $t_{i}^{F}$ is computed by the loss function, denoted as $\frac{\partial L}{\partial t_{i}^{F}}$. To enable the flow of error throughout the entire network, it is necessary to propagate the error for the single (first) step to all the associated spikes in the last layer. This process is divided into two steps: first, from the FS time $t_{i}^{F}$ to all steps $t_{i}^{L,n}$, and then from times $t_{i}^{L,n}$ to spikes $s_{i}^{L,n}$. According to the chain rule, the error of the spike in the output layer can be computed based on the error of the FS time and related gradients as follows:


(11) $$\begin{array}{l} \frac{\partial L}{\partial s_{i}^{L,n}}=\frac{\partial L}{\partial t_{i}^{F}}\overset{T}{\underset{m=1}{\sum }}\frac{\partial t_{i}^{F}}{\partial t_{i}^{L,m}}\frac{\partial t_{i}^{L,m}}{\partial s_{i}^{L,n}}. \end{array}$$

The key issue is to compute the gradient $\frac{\partial t_{i}^{F}}{\partial t_{i}^{L,n}}$ and $\frac{\partial t_{i}^{L,m}}{\partial s_{i}^{L,n}}$. Figure 3B illustrates the error assignment through these two steps.

#### 2.2.2. From FS times to temporal codes

First, for active neurons, as the FS time is given by the minimum of the temporal codes, the error of FS time is only related to the corresponding time step, which means that the derivative of the FS time with respect to temporal codes $t_{i}^{L,n}$ is 1 only when $t_{i}^{L,n}$ is the first-spike time. Specifically,


(12) $$\frac{\partial t_{i}^{ℱ}}{\partial t_{i}^{L,n}}=\{\begin{array}{cc} 1, & t_{i}^{L,n}=t_{i}^{ℱ},\\ 0, & otherwise. \end{array}$$

However, for inactive neurons, propagating the error through a single step will make the weights difficult to update. To address this issue, the error $\frac{\partial L}{\partial t_{i}^{F}}$ is assigned to all the other steps for inactive neurons, which means $\frac{\partial t_{i}^{F}}{\partial t_{i}^{L,n}}$ is always equal to 1. These two cases for active and inactive neurons are illustrated in Figures 3B(a, b).

Note that the strategy for inactive neurons has opposite effects on target and non-target neurons. This contrast arises from the fact that the loss function aims to minimize the first-spike time for target neurons while maximize it for non-target neurons. Hence, assigning error to all time steps is equivalent to minimizing (maximizing) the total firing time for target (non-target) neurons. Consequently, this strategy promotes the activation of dormant target neurons, while reinforcing the inactivity of non-target neurons that are already inactive.

#### 2.2.3. From temporal codes to spikes

The second gradient $\frac{\partial t_{i}^{L,m}}{\partial s_{i}^{L,n}}$ cannot be computed directly. In inference, temporal codes are exclusively linked to their corresponding spikes at a single step. The timestamp $t_{i}^{L,n}$ is generated from $s_{i}^{L,n}$ at the same step *n*. However, it is essential to involve spikes occurring around step *n* (*m*≠*n*) in the optimization of spike time at step *n*. During the learning process, the change of spike times results in the change of connections. If the optimization only focuses on a single spike at step *n* and its corresponding weights, the weight update would be inherently unstable. In the case of target neurons, the learning process optimizes not only the spike at step *n* but also the spikes and associated weights from earlier steps (*m*<*n*) to reduce output times. For non-target neurons, optimization should also involve spikes and related weights from later steps (*m*>*n*). Furthermore, since spike times can only change at most a few time steps at each iteration, the impact of error at step *n* diminishes as the time step is far away from *n*. This means that spikes closer to step *n* should receive a larger error assignment.

Therefore, a surrogate gradient needs to be designed to distribute error from $t_{i}^{L,m}$ to $s_{i}^{L,n}$. A Gaussian window is used to ensure a smooth and gradual weight update. In addition, the error of spikes should have an opposite sign to the error of times. The reason is that decreasing the spike time is equivalent to increasing the probability of spike firing in early steps, in other words, increasing the value of spikes from 0 to 1. Specifically, our approach is to distribute the error of time at step *m* to the spikes around it through a negative Gaussian window:


(13) $$\begin{array}{l} \frac{\partial t_{i}^{L,m}}{\partial s_{i}^{L,n}}=g\left(m-n\right), \end{array}$$

where *g*(*x*) is given by:


(14) $$\begin{array}{l} g\left(x\right)=-\frac{A}{2\pi \sigma }e^{-\frac{x^{2}}{2\sigma^{2}}}, \end{array}$$

where *A*>0 is the amplitude and $\sigma =\left\lfloor \frac{T}{D}\right\rfloor$ is the standard deviation, determined by the length of the sequence *T* and a constant factor *D*.

The width of Gaussian window is determined by 3-sigma limit and the length of whole sequence, *W* = min{6σ+1, *T*}. As depicted in Figure 3B(c), when the factor *D* increases, both the standard deviation σ and the window size *W* decrease, which means that the error assignment only focuses on the time steps surrounding the first spike. When *D* → ∞, σ → 0, and *W* → 1, the error is only assigned to the current step. On the contrary, when *D* → 0, σ → ∞, and *W*→*T*, the error is propagated to all the steps with approximately the same value, which is similar to the error propagation of the loss based on FR [see Figure 3B(d)]. The parameters *A* and *D* are determined empirically. The value of *A* should not be too small, usually around 2*T*, otherwise the training is slow with a deteriorated performance. The values of *D* and window size *W* have a significant impact on the performance which are discussed further in Section 3.5.

#### 2.2.4. Surrogate gradient descent training through spikes

After propagating error from FS times to spikes in the output layer, the error can be propagated through spikes in the rest of the network. To solve the non-differentiability of the spike function, a surrogate gradient (Zenke and Ganguli, 2018) is used to estimate the derivative of postsynaptic spikes with respect to membrane potential. Specifically, the gradient of the step function in Eq. (5) is estimated using a fast sigmoid function in the backward pass:


(15) $$\begin{array}{l} f^{\prime }\left(U\right)\approx \frac{1}{{\left(1+\rho |U-\theta |\right)}^{2}}. \end{array}$$

Having the error assignment from FS times to spikes and surrogate gradients of spike function, the overall backpropagation pipeline can be constructed. As shown in Figures 3A, B, the error flows from time $\frac{\partial L}{\partial t^{L,n}}$ to spike $\frac{\partial L}{\partial s^{L,n}}$ in the last layer, given by Eqs (12), (13). In each layer, the error of spikes $\frac{\partial L}{\partial s^{l,n}}$ is propagated to membrane potential $\frac{\partial L}{\partial U^{l,n}}$ through the surrogate gradient in Eq. (15) and then the error of synaptic current $\frac{\partial L}{\partial I^{l,n}}$ can be calculated. Finally, the derivative of error with respect to weights ***W***^*l*^, ***V***^*l*^ in each layer are calculated by taking the derivative of Eq. (2):


(16) $$\begin{array}{l} \frac{\partial L}{\partial W^{l}}=\overset{T}{\underset{n=1}{\sum }}\frac{\partial L}{\partial I^{l,n}}\frac{\partial I^{l,n}}{\partial W^{n}}=\overset{T}{\underset{n=1}{\sum }}\frac{\partial L}{\partial I^{l,n}}s^{l-1,n}, \end{array}$$

and


(17) $$\begin{array}{l} \frac{\partial L}{\partial V^{l}}=\overset{T}{\underset{n=1}{\sum }}\frac{\partial L}{\partial I^{l,n}}\frac{\partial I^{l,n}}{\partial V^{n}}=\overset{T}{\underset{n=1}{\sum }}\frac{\partial L}{\partial I^{l,n}}s^{l,n}. \end{array}$$

### 2.3. Strategies facilitating training based on FS timings

In addition to the computation of gradients, parameter initialization is also crucial to the training of SNNs. Time constant τ, threshold θ, temporal resolution Δ*t*, the length of the sequence *T*, and weight initialization have a great impact on the results. In addition, training based on FS timings is more challenging because only focusing on the first spike leads to more inactive neurons during training. Thus, apart from the error assignment from time-to-spike described in Section 2.2.1, other strategies can be used in parameter settings to facilitate the training process.

#### 2.3.1. Different time constants and thresholds for feature extraction and decision

**Figure 4:**
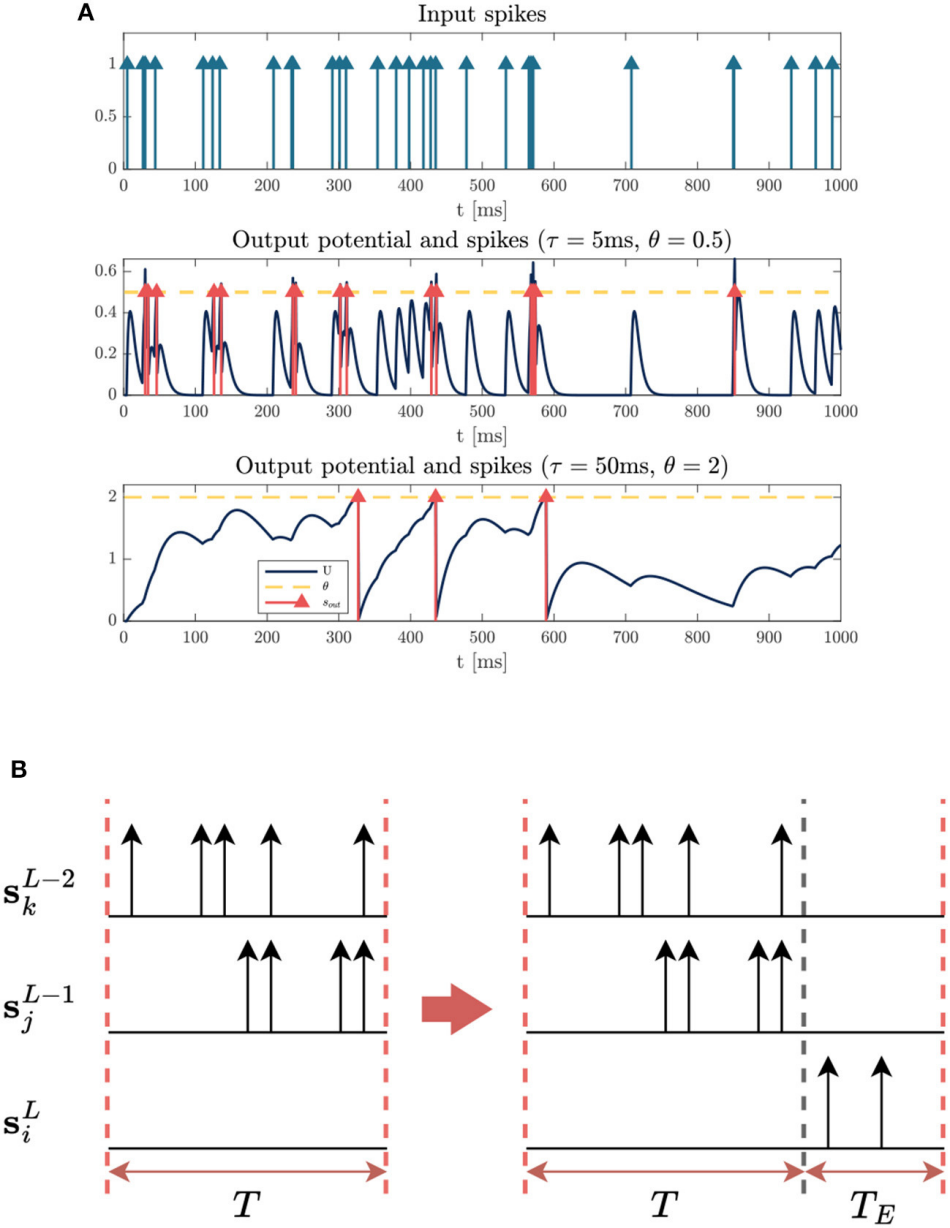
Illustrations of strategies to facilitate training with the FS loss. **(A)** Dynamics of neurons with different time constant τ and threshold θ. **(Top)** Input spike sequence (all the weights are equal to 1). **(Middle)** The neuron with τ = 5*ms*, θ = 0.5 responds rapidly to local features. **(Bottom)** The neuron with large parameters τ = 50*ms*, θ = 2 keeps a longer memory of input signals. **(B)** Illustration of how an active neuron with a large time delay can be seen as inactive. We can see that there exists a delay for spikes propagating down the network, causing the neuron to fail to fire a spike within the input window. By extending the time window with an empty sequence, the inactive neuron becomes active.

An appropriate time constant τ and threshold θ can enhance the performance of the system, which determine the firing rate and neuron activity in the system. Empirically, we found that smaller values of τ and θ in the first few layers but larger values for the final layers leads to better performance on FS coding, which is consistent with our intuition. Figure 4A illustrates the responses of neurons with different τ and θ to the same input sequences. The neuron with a small value of τ has a short memory due to the rapid decay of its membrane potential. Meanwhile, a small θ is used to maintain a high firing rate, thereby facilitating the transmission of sufficient information. Therefore, small values of τ and θ are well-suited for capturing local features in the initial layers.

On the contrary, the final layer requires a longer delay to make a correct decision after enough information is accumulated. We can see from Figure 4A that large values of τ and θ can help keep a longer memory of previous stimuli, ensuring the target output neuron fires the first spike after receiving enough spikes from previous layers. Experiments in Section 3.3 confirm that an appropriate longer time delay of the first spike leads to higher accuracy of prediction.

#### 2.3.2. Extension of time window with empty sequences

One of the challenges in training FS-coded model is dealing with inactive neurons. Having too many inactive neurons in the network will stop the gradient flow and hamper the update of weights, making the training more difficult. In the output layer, particularly, there is no precise timing for the optimization of inactive neurons. However, we found that some of them are only inactive due to delay between layers which leads to spikes beyond the observed time window, as visualized in Figure 4B.

We thus extend the input window by appending an empty sequence to the end during training. We can see from Figure 4B that the empty sequence allows the output neuron to fire a later spike outside the original window. The length of empty sequence *T*_*E*_ is determined by time constant τ of the last layer empirically. Usually, a network with a larger time constant is more likely to exhibit a longer delay, therefore, a larger *T*_*E*_ should be used in that case. Note that this strategy is only used to facilitate training, and it is useful to enhance accuracy when τ in the last layer is very large and the original window size is relatively small. The downside of this approach is a higher computation workload and a longer delay of the target FS (see Section 3.3). For the experiments in Section 3, we set *T*_*E*_ = 0 unless otherwise specified.

## 3. Results

### 3.1. Experimental settings

We compare models using FS and FR coding schemes on classification tasks. We avoid using unrealistic datasets, such as N-MNIST (Orchard et al., 2015) and CIFAR10-DVS (Li et al., 2017), in which the first spike is not meaningful because events are generated by moving neuromorphic device around static images and there are no significant temporal differences in their sequences (Iyer et al., 2021). Instead, we test our model on realistic datasets in which important information is encoded in spike timings. For visual datasets DVSGesture (Amir et al., 2017) and DVSPlane (Afshar et al., 2019), events are generated by real cameras capturing dynamic scenes. For auditory datasets N-TIDIGITS (Anumula et al., 2018) and SHD (Cramer et al., 2022), spikes are derived by converting existing datasets using a neuromorphic device or a realistic simulator. As shown in Figure 1, the temporal structures of event sequences in these datasets are different. For example, auditory signals in SHD and N-TIDIGITS are non-repetitive and the temporal complexity is much higher than visual datasets, since spatial information is crucial in the prediction of visual data. In addition, signals in DVSGesture contain repetitive information, in which a person performs the same gesture several times. By contrast, DVSPlane exhibits more temporal complexity since the movement is not periodic. We observed that FS and FR models exhibit different behavior on these signals with varying temporal structures.

In our settings, the architecture and parameters vary among different tasks. Note that our aim is to make a comparison between FR and FS codings rather than seeking highest accuracy, hence relatively simple architectures are used in our study. The notation of architecture remains consistent throughout the article. For example, [2,32,32]-32C5S2-P2-64C3-FC128(R)-10 represents a network with a [2,32,32] input, where the first convolutional (C) layer contains 32 kernels (5 × 5) with a stride (S) of 2, followed by a max pooling (P) (2 × 2) and 64 convolutional kernels (3 × 3) with a stride of 1 (default), and finally a fully connected (FC) layer with 128 neurons with 10 recurrent (R) connection and output classes. Weights are all initialized using Xavier uniform distribution, and the Adam optimizer with a weight decay of 1*e*^−4^ is used. The parameter of surrogate gradient ρ is set to 5 in all the cases. Notations for learning rate, batch size, and the number of epochs are η, *B*, and *N*_*ep*_, respectively. We denote time constant and threshold as τ_1_ and θ_1_ for those used in feature extraction, and as τ_2_ and θ_2_ for those used in decision process. In a convolutional SNN, τ_1_/θ_1_ are used in convolutional and pooling layers, and τ_2_/θ_2_ are used in FC layers. For FC architecture, τ_1_/θ_1_ are used in hidden layers while τ_2_/θ_2_ are used in the output layer. In our experiments, τ_1_/θ_1_/θ_2_ are determined empirically to obtain the optimal results. The value of τ_2_ affects time delay and accuracy significantly, whereas the value of θ_2_ does not have a great impact on the performance when τ_2_ is fixed. We, therefore, focus on τ_2_ and test different values with τ_2_ = μτ_1_. Further details are introduced in the following and in Table 1.

**Table 1:** Hyperparameters for different tasks.

	**Neuron**				**FS**					**FR**	**Training**		
	***Δt***	**τ_1_**	**θ_1_**	**θ_2_**	**α_0_**	**λ**	**β_0_**	***A***	***D***	**α_1_**	**η**	***N*_*ep*_**	***B***
DVSGesture	10 ms	50 ms	0.5	1	0.1	0.01	0.02	300	4	10	1*e*^−4^	40	16
SHD	10 ms	50 ms	5	10	0.2	0.01	0.02	200	16	20	1*e*^−3^	80	128
N-TIDIGITS	5 ms	25 ms	1	2	0.1	0.01	0.02	500	16	20	1*e*^−3^	200	128
DVSPlane	2 ms	10 ms	0.5	2	0.1	0.01	0.02	500	8	15	3*e*^−4^	50	16

#### 3.1.1. DVSGesture dataset

DVSGesture (Amir et al., 2017) is captured by DVS128 camera, including 11 hand gestures recorded under three different lighting conditions. Since gestures in the last class are random, we only take the first 10 classes. Recordings are split into 1,078 and 264 samples for training and testing, respectively. Each sequence is around 6 s, we clip 1.2 s in training and 2.5 s for testing, with a temporal resolution Δ*t* = 10 ms. The frame size is 128 × 128, which is downsampled by 4 for the input. The architecture is [2,32,32]-64C3-128C3-P2-128C3-P2-FC128(R)-10.

#### 3.1.2. SHD dataset

Spiking Heidelberg Dataset (SHD) is a dynamic audio dataset generated using Lauscher, an artificial cochlea model, including 20 classes of spoken digits from 0 to 9 in both German and English languages. A total of 7,736 samples are used for training and 2,264 samples for testing. Each sequence is around 1 s. We clip 0.8 s and 1 s in training and testing, respectively, and Δ*t* = 10 ms. The architecture is 700-FC256(R)-20, in which the time constant in the hidden layer is initialized with τ_1_ but related variables α, β are trainable using the method by Perez-Nieves et al. (2021).

#### 3.1.3. N-TIDIGITS dataset

N-TIDIGITS Dataset (Anumula et al., 2018) transforms TIDIGITS dataset into spikes with the dynamic audio sensor, CochleaAMS1b. It includes 11 classes of spoken numbers 0–9 and a word “oh.” The length of sequences is around 0.08–2.46 s but most of the sequences are <1.2 s. Δ*t* = 5*ms* and *T* = 250 are used in all sequences. Training and testing datatsets include 2,463 and 2,486 samples, respectively. The architecture is 64-FC256(R)-FC256(R)-11, in which time constant in hidden layers are trainable.

#### 3.1.4. DVSPlane dataset

DVSPlane dataset (Afshar et al., 2019) is captured by an asynchronous time-based image sensor (ATIS). Here, four different airplane models are dropped free-hand from varying heights and distances in front of the camera. The length of sequences is 242 ± 21 ms. We set Δ*t* = 2 ms and *T* = 100 in training, and *T* = 120 in testing. The 800 samples are split into 640 and 160 samples for training and testing. The image with a size of 304 × 240 is downsampled by 4 as input. The architecture is [2,76,60]-32C5S2-64C3-P2-128C3-P2-FC256(R)-4.

#### 3.1.5. Evaluation

The results are compared in terms of accuracy, time delay, and spike count. If not specified, the accuracy of the FS or FR model is evaluated in a manner that is consistent with its training.

**Figure 5:**
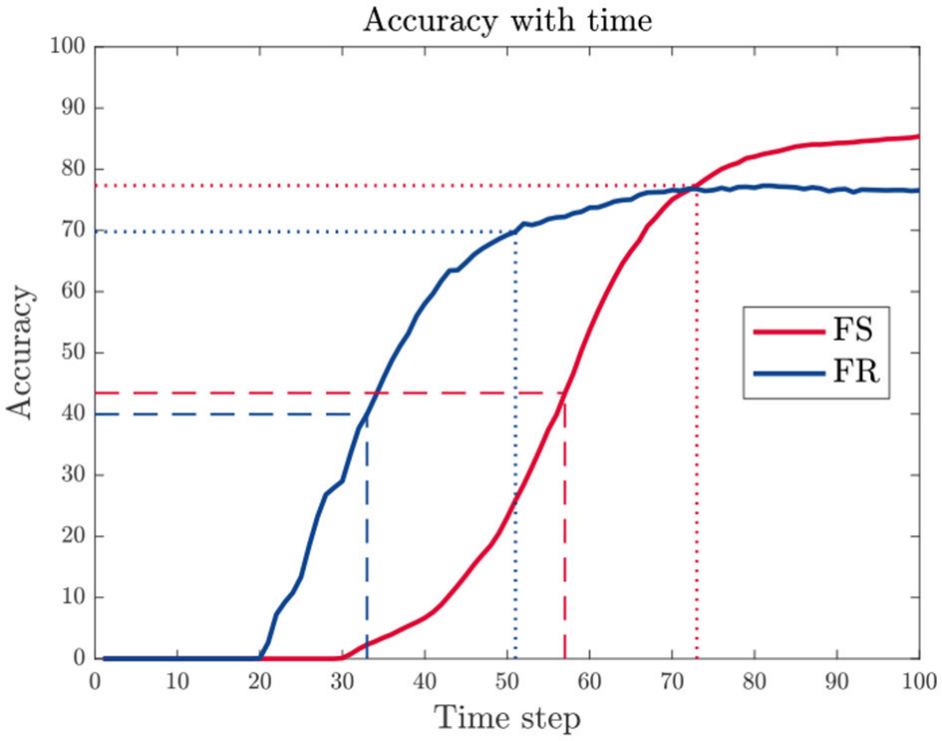
An example of accuracy variation with increasing time window size. The time delay *t*_*d*_ is evaluated using the time when the accuracy reaches 50% (dashed line) or 90% (dotted line) of the peak value within the given time window.

As shown in Figure 5, the accuracy increases with longer time window. We, therefore, evaluate time delay as time when reaching 50 or 90% of the peak accuracy within the given time window, denoted as *t*_*d*_(50%) and *t*_*d*_(90%), respectively.

In terms of energy consumption, many studies calculate the number of synaptic operations as a measure. However, these metrics are usually employed when comparing power consumption between SNNs and ANNs, or between different architectures. They usually involve multiplying the number of connections by spikes (Wu et al., 2022), or factoring in firing rate, time step, and FLOPs (Zhou et al., 2023). Nonetheless, in our settings, we only change the coding scheme in the output layer while maintaining the same architectures. In addition, FS coding scheme does not introduce additional operations during inference. Therefore, energy consumption mainly depends on the spike count in the system. Here, we use the average number of spikes per neuron in the system (denoted as *N*_*s*_) to evaluate power consumption.

Furthermore, to test the required number of spikes for correct classification, an optional constraint for the spike count is added in the loss function to constrain the total number of spikes in the system, i.e., $L_{s}=\lambda_{s}|N_{s}-{Ñ}_{s}|$, where Ñ_*s*_ is the target average spike count per neuron and λ_*s*_ is a constant value. The range of λ_*s*_ extends from 0.1 to 3, and we empirically adjusted it for various FS and FR models. Results under different spike count constraints are discussed in Sections 3.3 and 3.4.

### 3.2. Results overview

We compare the results of models using FS and FR codings, respectively, on the four datasets. During the training of FS models, an empty sequence is added with lengths *T*_*E*_ = 40 and *T*_*E*_ = 20 for DVSGesture and SHD data sequences, respectively. As shown in Table 2, we can see that the FS model demonstrates comparable performance with the FR model. We also tested different time constant τ_2_ for decision layers. For FS models, a larger τ_2_ leads to a higher accuracy on both metrics, but the time delay *t*_*d*_ also increases. In contrast, for FR models, τ_2_ does not affect the accuracy significantly. It seems that a longer first-time latency encodes more information. The relationship between accuracy and time delay is further discussed in Section 3.3. In addition, the response time *t*_*d*_ of the FR model is shorter than the FS model overall. Note that the disparity between time delays for FR and FS models is large when reaching 50% accuracy, but the gap is reduced when achieving 90% of the best performance in some cases, especially for models with small time constants.

**Table 2:** Comparison of FR and FS models with different time constant τ_2_ for decision layers (τ_2_ = μτ_1_) in terms of accuracy (Acc), spike count (*N*_*s*_), and time delay (*t*_*d*_).

		**Trained using FR**				**Trained using FS**			
	μ	**Acc(%)**↑	*N*_*s*_↓	*t*_*d*_(50%)↓	*t*_*d*_(90%)↓	**Acc (%)**↑	*N*_*s*_↓	*t*_*d*_(50%)↓	*t*_*d*_(90%)↓
DVSGesture	1	94.0	10.0	**36.2**	58.6	90.1	10.2	38.2	**56.8**
		4	94.2	9.5	41.6	58.8	89.7	9.6	41.8	59.0
		12	**95.1**	11.0	45.4	60.2	92.8	**8.9**	61.6	77.6
SHD	1	79.3	26.8	**28.8**	**46.2**	78.3	24.9	33.8	47.2
		4	78.8	31.0	30.8	47.2	85.5	12.6	48.4	67.0
		12	77.8	35.7	33.0	49.4	**87.6**	**9.0**	58.0	74.8
N-TIDIGITS	1	88.2	10.6	**74.4**	133.0	87.6	12.1	88.0	**129.4**
		4	88.7	11.4	76.6	136.8	**89.3**	5.4	94.4	137.8
		8	88.1	11.5	80.6	139.4	**89.3**	**5.2**	98.2	144.0
DVSPlane	1	83.0	**1.3**	57.6	84.4	87.6	2.0	**52.0**	**65.2**
		4	85.4	1.5	68.0	85.8	90.5	2.0	54.6	67.6
		8	89.3	1.8	67.8	82.8	**92.7**	1.9	62.0	77.0

The results are the average of 5 trials. The bold values represent the best results obtained when comparing the FR and FS systems for each dataset.

One benefit of using FS coding is the reduction in the number of spikes, thereby leading to better energy efficiency. We can see from Table 2 that the number of spikes *N*_*s*_ in an FS system is usually smaller than the number in an FR system, especially for SHD and N-TIDIGITS classification. Further experiments demonstrate that FS models are robust with fewer spikes, as shown in Section 3.4.

**Figure 6:**
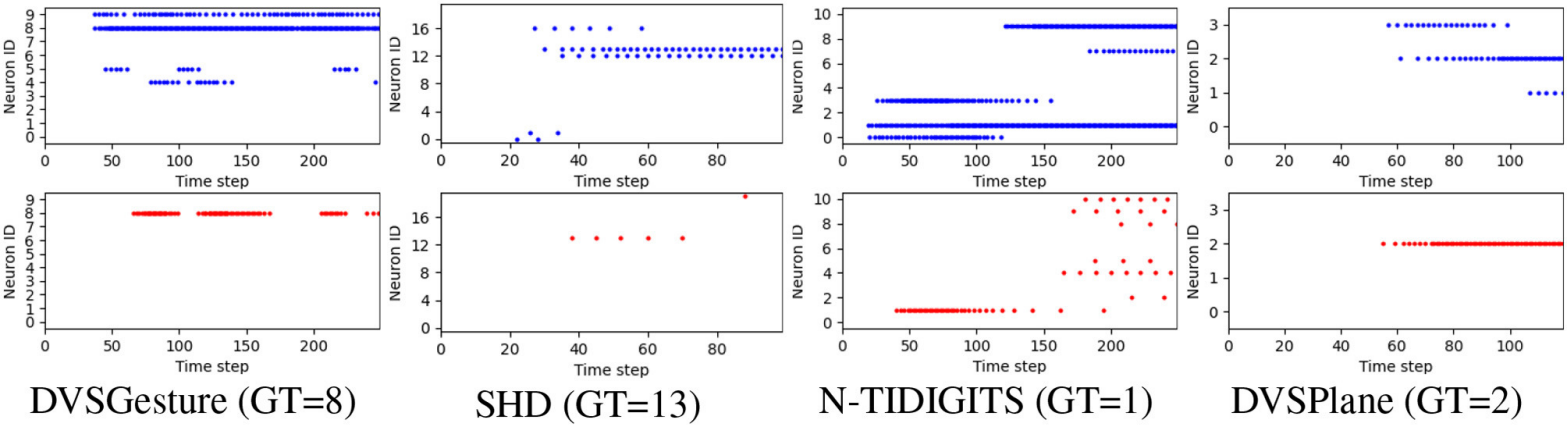
Representative output spike raster plots of correct prediction using models trained with FR (above, blue) and FS (bottom, red), respectively, τ_2_ = 4τ_1_ in all the models. Training with FR leads to faster responses but non-target neurons fire more spikes, while the FS coding leads to lower firing rate and reduces the likelihood of misclassification because non-target neurons fire fewer spikes.

Furthermore, we generated the output spike raster plots to analyze the neuronal activities of the FS and FR systems. As depicted in Figure 6, non-target neurons of an FS model fire fewer spikes than an FR model, which reduces the likelihood of misclassification based on FR. The reason is that the first time of the non-target neuron is optimized to the end of the sequence, which reduces the probability of firing in the whole sequence significantly. This phenomenon allows the FS model to make decisions based on FR as well. Further results on data sequences with different temporal structures are analyzed in Section 3.5.

To validate the effectiveness of FS coding, further experiments are conducted on models using adaptive LIF neurons in Section 3.6.

### 3.3. Trade-off between accuracy and time delay

During training, we found that it is easier to train FS models when time constant is relatively large and a proper empty sequence is added. Results also indicate that there is a trade-off between accuracy and response time. The two main factors that affect time delay, time constant and length of empty sequences, are analyzed in the following.

#### 3.3.1. Time constant

As observed from Table 2, the FS model with a larger time constant τ_2_ in the last layers leads to higher accuracy and fewer spikes in the system overall, but at a cost of longer time delay.

#### 3.3.2. Time window and empty sequence

Another factor that affects time delay is the length of the input time window, which is determined by the original length of data *T* and the length of added empty sequence *T*_*E*_. First, we used a fixed *T* and tested different *T*_*E*_ values on the DVSGesture dataset. The model with τ_2_ = 12τ_1_ is tested because the empty sequence is added only when the original window size *T* is relatively small for a long time delay (large τ_2_). As shown in Table 3, an increasing *T*_*E*_ leads to a longer time delay and a higher overall accuracy.

**Table 3:** Results of DVSGesture classification with different length of data sequence *T* and added empty sequence *T*_*E*_ (*N*_*ep*_ = 50).

***T*** = 120, τ_**2**_ = 12τ_**1**_				***T***_*****E*****_ = 0, τ_**2**_ = τ_**1**_			
*T*_*E*_	**Acc(%)** ↑	*t*_*d*_(50%)↓	*t*_*d*_(90%)↓	*T*	**Acc (%)** ↑	*t*_*d*_(50%)↓	*t*_*d*_(90%)↓
0	92.4	**59**	**82**	60	81.1	**29**	**35**
20	93.6	64	95	80	86.0	35	44
40	93.6	65	97	100	88.3	36	48
60	**93.9**	66	99	120	90.9	38	53
80	**93.9**	77	114	140	**89.4**	48	82

A larger window size *T*+*T*_*E*_ leads to higher accuracy but a longer time delay. The bold values represent the best results in each column.

Furthermore, different length *T* of input data is tested for the model with τ_2_ = τ_1_, where *T*_*E*_ = 0 for all the trials. The second column in Table 3 shows that the accuracy is higher with a larger window size.

#### 3.3.3. Accuracy vs. time delay under spike count constraints

**Figure 7:**
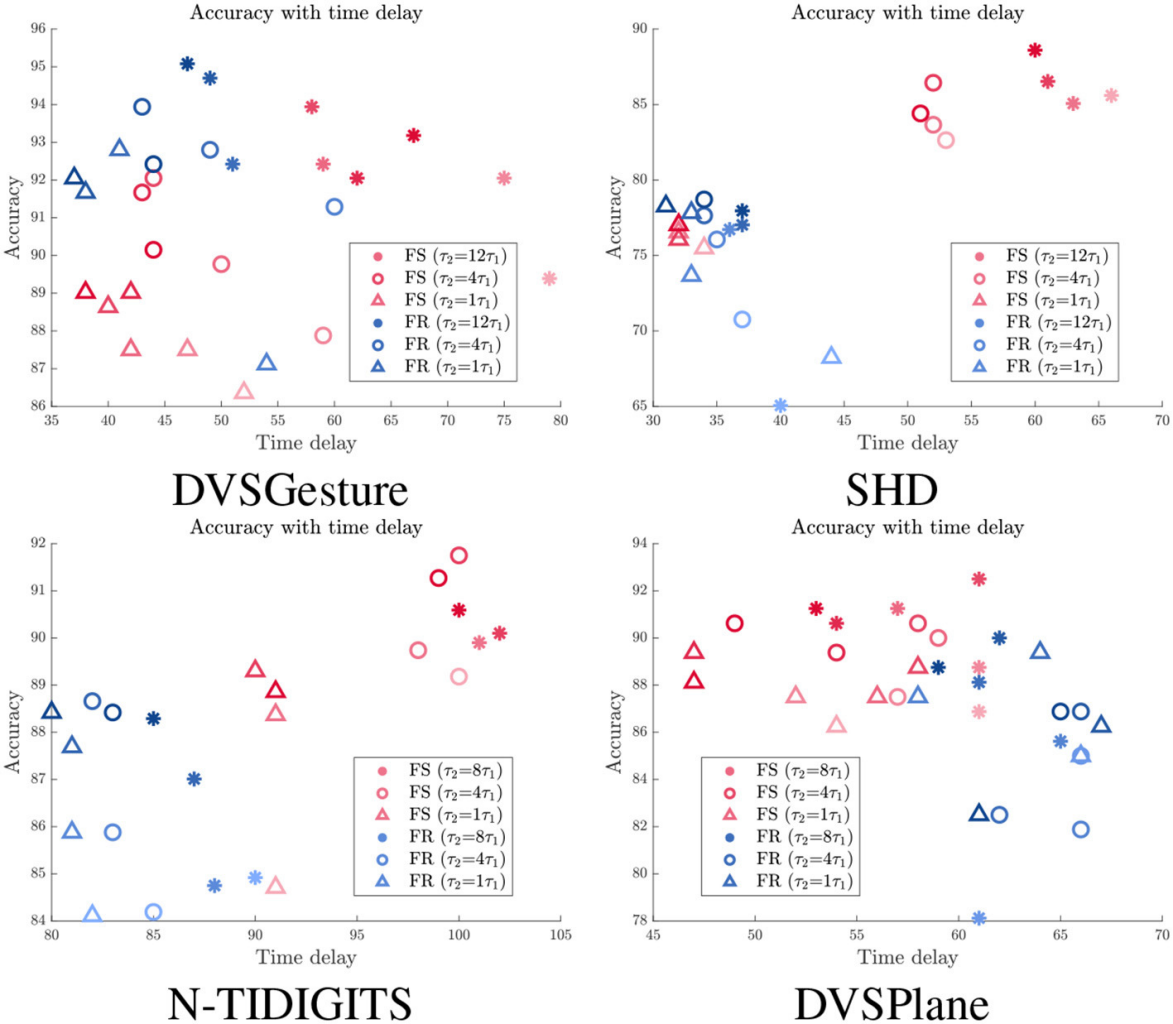
Relationship between accuracy and time delay *t*_*d*_ for models with different time constants τ_2_ under different spike count constraints. Red: FS models. Blue: FR models. Darker colors indicate results with larger target average spike count Ñ_*s*_.

We further imposed different spike count constraints to FS and FR models with different time constant τ_2_. The time delay and corresponding accuracy of each trial are displayed in Figure 7. We can see that the time delay is mainly determined by time constant. FR models usually exhibit shorter time delay than FS models, except for DVSPlane. It is clear that FS models for SHD and N-TIDIGITS datasets achieve higher accuracy with longer time delay. The spike raster plots of SHD and N-TIDIGITS classifications in Figure 6 also indicate that FS models achieve higher accuracy with longer time delays and fewer spikes, while FR models respond faster but more spikes produced by non-target neurons interfere with the classification. For DVSGesture and DVSPlane datasets, although the relationship between FS and FR models is not obvious, the best FS results are generated when we have larger temporal latency.

It is also worth noting that there are points with longer *t*_*d*_ but lower accuracy under the same time constant. These points represent the models with smaller number of spikes, which highlights that time delay tends to be longer with fewer spikes. Overall, fewer spikes in the system usually lead to a lower accuracy and longer time delay. The relationship between accuracy and spike count is further discussed in Section 3.4.

To summarize, appropriate time delay ensures that the first spike makes a correct decision. In other words, the first spike encodes more information with a longer time delay. However, a model with a larger τ_2_ has a risk of lacking sufficient spikes to make a decision due to a lower output firing rate. In addition, it becomes more difficult to improve the accuracy as the cost of time delay increases, because the accuracy is not only determined by time delay but also limited by the model itself and other factors.

### 3.4. Energy efficiency

In SNNs, the power consumption mainly depends on the mean spike activity and the number of synaptic operations (Parameshwara et al., 2021). When deploying SNNs on neuromorphic hardware such as the Intel Loihi (Davies et al., 2018), reducing the number of spikes in the systems could leads to gains in energy efficiency.

Table 2 illustrates that FS systems produce fewer spikes than FR systems overall, especially on SHD and N-TIDIGITS datasets. DVSGesture and DVSPlane classifications with CNN architecture generate approximately the same number of spikes in both systems. The FS system with a larger time constant is usually more energy efficient, while the FR system generates fewer spikes with a smaller τ_2_.

**Figure 8:**
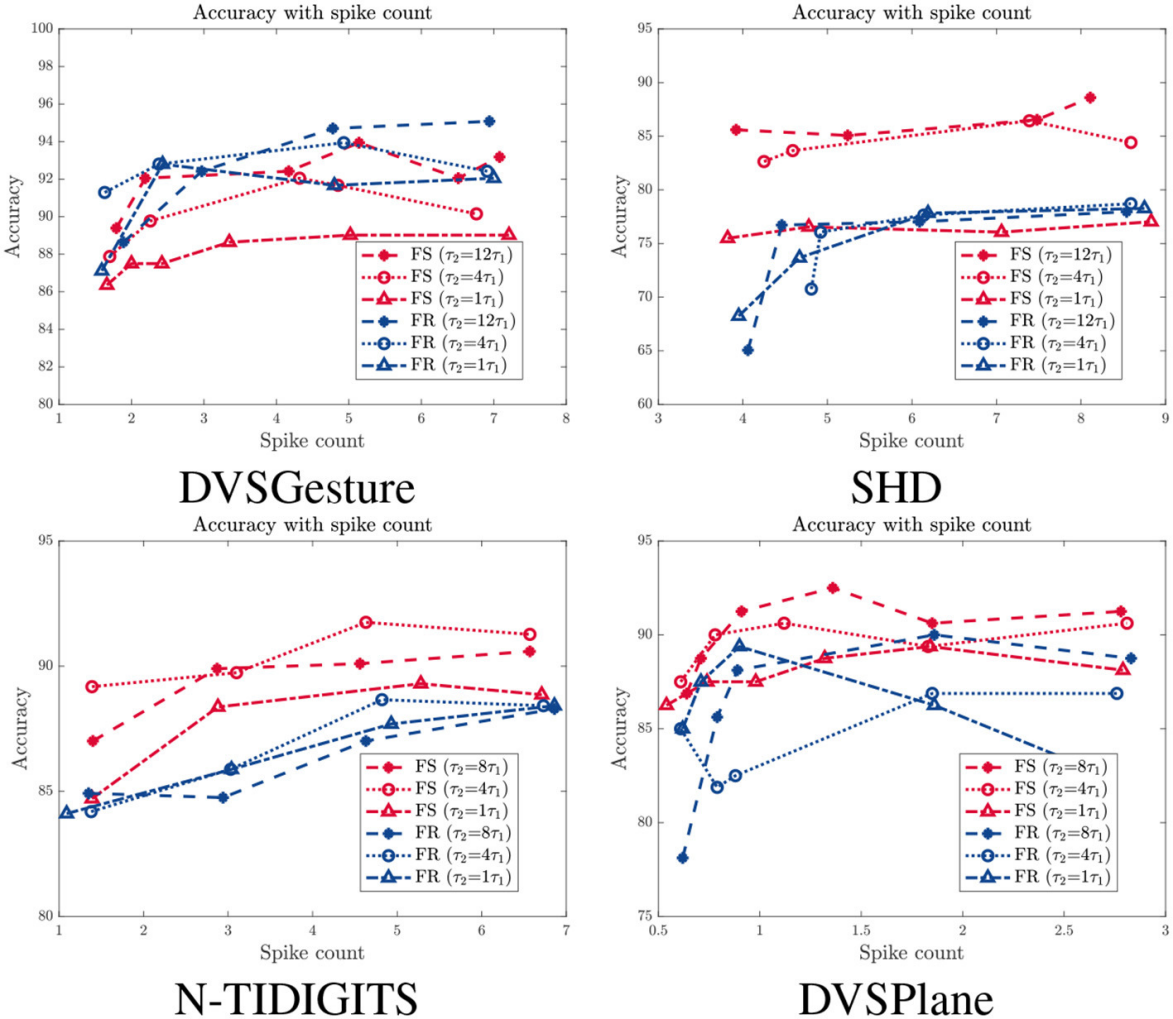
Accuracy with different spike count *N*_*s*_ for models with different time constant τ_2_. Red: FS models. Blue: FR models.

As mentioned in Section 3.3, reducing the number of spikes through a spike count constraint results in a decline in performance. Figure 8 presents the relationship between accuracy and average spike count *N*_*s*_ for models with different time constants τ_2_, in which red and blue curves represent results of FS and FR models, respectively. We can see that the accuracy of FR models decreases significantly overall, while the FS modes is more stable with fewer spikes, especially for SHD and N-TIDIGITS tasks. For DVSGesture dataset, the original spike counts of FS and FR models are close, so accuracy of both models drops as the spike count decreases. Note that the original *N*_*s*_ of FS models is larger than FR models in DVSPlane classification, but the accuracy of FR models decreases more significantly. In addition, the FS model with a large τ_2_ is more robust to reduced number of spikes. Overall, FS models with a large time constant are more energy efficient and more robust to the spike count constraint.

### 3.5. Performance and behavior on event sequences with different temporal structures

From Table 2, we can see that the performance varies on different datasets. Overall, the FS system model outperforms the FR model on SHD/N-TIDIGITS/DVSPlane datasets, whereas its performance on DVSGesture task is relatively inferior. These differences are due to different temporal structures in the input sequences. As illustrated in Figure 1, in the DVSGesture dataset, the same gesture is repeated several times. The target neuron is expected to keep firing and make a consistent decision. In contrast, the data pattern of the audio data is non-repetitive. The frequencies of spoken digit numbers are changing over time within a short period, so that the neuron do not have to keep firing after a prediction has been made. Data sequences in DVSPlane are also non-repetitive, but the spatial features do not change significantly during the dropping of an airplane.

#### 3.5.1. Neuron activities

**Figure 9:**
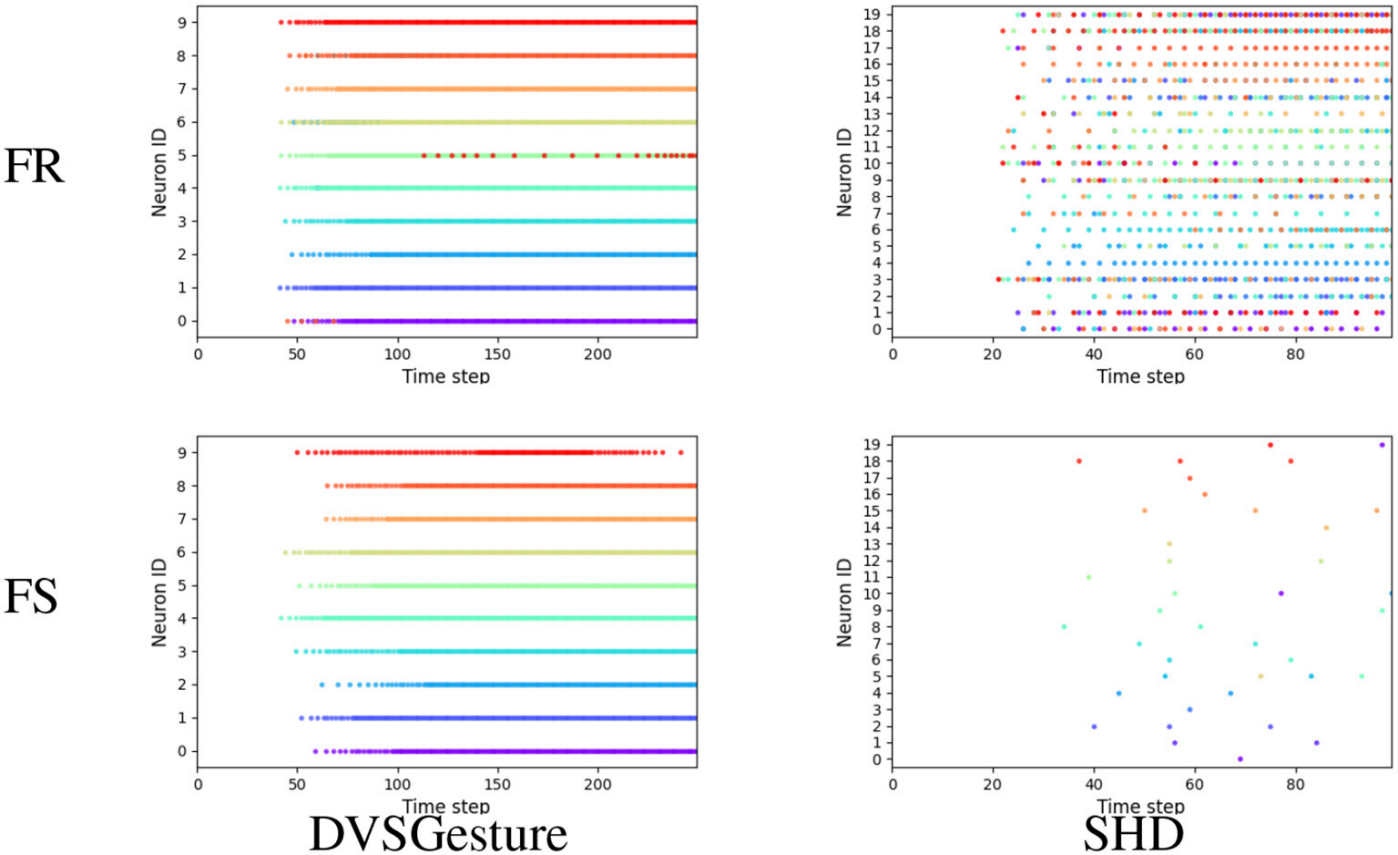
Output spike raster plots of FR and FS systems for different types of data. Each figure illustrates the output spikes generated in response to various input signals that belong to different classes. Each color corresponds to the output spikes from a single trial. We can observe that the neuronal activities of FS and FR systems are significantly different on the SHD dataset with rich temporal structures, whereas for the signals with temporal repetition (DVSGesture), the output spike patterns of both systems are similar.

To better compare the firing patterns between FS and FR models, apart from Figure 6, we aggregated all the output spikes generated in response to different input signals for each class in a single raster plot, as shown in Figure 9. Each color corresponds to the output spikes from a single trial. An ideal case is that each neuron generates spikes of only one color, such as FS-DVSGesture in Figure 9. This indicates that only the target neuron is active while the other neurons remain inactive. On the other hand, mixed colors (such as FR-SHD) indicate that the non-target neurons fire more spikes thereby affecting classification.

As shown in Figures 6, 9, it is notable that FS models exhibit distinct neuronal behavior on different types of data. For the visual signals in DVSGesture and DVSPlane, FS models produce periodic firing pattern similar to those produced by FR models, in which the target neuron keeps firing to consistently make a prediction. However, for the SHD data sequences without temporal repetition, FS systems generate much fewer spikes than FR systems and the target neuron almost stops firing after the classification has been made.

The distinct neuronal activities of SHD also illustrate why FS coding can outperform FR coding even though the FS coding makes decisions based on only a portion of the given input spikes. First, FS models usually exhibit a longer temporal delay than FR models. This highlights that ensuring high accuracy demands sufficient input information. The time delay indicates the minimum required information for accurate decision-making. From FS-SHD in Figure 9, the time delay varies notably across different trials. This indicates that different lengths of input data are necessary for making correct decisions. While FR coding utilizes entire given input data, it can include redundant information. As shown by FR-SHD in Figure 9, output neurons start firing at approximately the same time across different trials, even when the available information is not enough for correct decision-making. Non-target neurons generate more spikes that disrupt the decision process, leading to a lack of precision in decision-making.

The unique output spike pattern suggests that FS models is also capable of achieving accurate classifications based on FR. In Table 4, both FS and FR accuracies are tested on the two types of models. Results demonstrate that FS models performs well on FR and sometimes even better than FR models, although it is trained based on FS timings. However, FR models struggle to predict accurately based on FS.

**Table 4:** The accuracy of FS and FR models with different time constants τ_2_ for decision layers (τ_2_ = μτ_1_).

		**Trained using FR loss (%)**		**Trained using FS loss (%)**	
	μ	**Acc (FS)**↑	**Acc (FR)**↑	**Acc (FS)**↑	**Acc (FR)**↑
1	72.3	94.0	90.1	93.7
	4	81.0	94.2	89.7	94.3
	12	82.6	**95.1**	**92.8**	94.6
SHD	1	34.3	79.3	78.3	70.5
		4	39.4	78.8	85.5	79.1
		12	42.8	77.8	**87.6**	**79.4**
N-TIDIGITS	1	47.9	88.2	87.6	75.6
		4	47.4	**88.7**	**89.3**	87.0
		8	51.0	88.1	**89.3**	88.0
DVSPlane	1	69.5	83.0	87.6	88.5
		4	76.5	85.4	90.5	**90.3**
		8	82.0	89.3	**92.7**	90.0

The results are the average of 5 trials. Both accuracy based on FS and FR are tested. Acc (FS) and Acc (FR) indicate accuracy when testing using FS and FR, respectively. The bold values represent the best results obtained when comparing the FR and FS accuracy for each model.

#### 3.5.2. Gaussian window size in error propagation

Another interesting observation is that the choice of Gaussian window size in the error propagation is related to the data type. We observed that repetitive visual data achieves better performance with a larger Gaussian window *W* (i.e., with a smaller *D*), while non-repetitive audio sequences prefer a smaller value of *W*. Specifically, we obtain optimal results using *D* = 16 for SHD and N-TIDIGITS, and *D* = 4, *D* = 8 for DVSGesture and DVSPlane, respectively. A larger window size in error propagation means that the error of FS times is propagated to a wider time range and more spikes are optimized. As a result, the firing patterns are more similar to rate coding with a higher firing rate but the precise timing of spikes is lost. On the contrary, with a smaller window size, the error is propagated to fewer spikes where the precise timing is emphasized. Table 5 shows the results of the FS model on repetitive (DVSGesture) and non-repetitive (SHD) data with different Gaussian window size *W*. As *W* decreases, the firing rate of the target neuron decreases since fewer spikes are optimized, leading to a drop in the FR accuracy. Nevertheless, there are distinct behaviors in the FS accuracy of repetitive and non-repetitive signals. The FS accuracy of repetitive data follows a similar trend to FR accuracy, but it improves with a smaller window on non-repetitive data, as the precise timing is more important in this case.

**Table 5:** Comparison of results on repetitive visual data (DVSGesture) and non-repetitive audio data (SHD) with different Gaussian window size *W* in error assignment.

**DVSGesture (**τ_**2**_ = 4τ_**1**_**)**				**SHD (**τ_**2**_ = 4τ_**1**_**)**			
*D*	*W*	**Acc (%)(FS)** ↑	**Acc (%) (FR)** ↑	*D*	*W*	**Acc (%) (FS)** ↑	**Acc(%) (FR)** ↑
4	120	**89.0**	**94.3**	4	120	82.9	**81.8**
8	91	88.6	93.2	8	73	84.5	80.3
12	61	86.7	88.6	16	37	**85.6**	77.3
16	46	88.6	90.2	32	19	85.5	76.5

As the window size *W* decreases, the FR accuracy drops due to a lower firing rate. The FS accuracy of repetitive signals experiences a decline as well, whereas that of non-repetitive signals sees an opposite trend where the precise timing is important. The bold values represent the best results in each column.

We can, therefore, conclude that FS coding is better suited for the classification of non-repetitive dat and when precise timing of the first spike is required. FR neurons should be used for repetitive signals or the cases in which a consistent and stable prediction is required.

### 3.6. Results of models using AdLIF neurons

To further validate the effectiveness of the FS coding, we conducted further experiments by replacing CUBA-LIF neurons with adaptive LIF (AdLIF) neurons (Bittar and Garner, 2022) in hidden layers. We tested the FC models for the SHD and NTIDIGITS tasks and recurrent connections are removed. Note that CUBA-LIF neurons with fixed parameters are still used in the output layer, because we found that this type of neuron can enjoy longer delay for accurate decision-making. We only tested models with large time constant in the output layer and utilized batch normalization and dropout strategy in hidden layers to obtain the best accuracy. Detailed parameter settings are listed in Appendix.

Table 6 presents comparison results between FS and FR models utilizing AdLIF neurons. The same conclusion can be drawn from these results as those derived from models using CUBA-LIF neurons. The FS coding leads to higher accuracy and superior energy efficiency (fewer spikes) than FR coding on data sequences with rich temporal structures. On the other hand, FS models exhibit longer time delay compared to FR models, but the gap is reduced as the accuracy reaches 90% of its peak value. Furthermore, compared to the results of SHD and NTIDIGITS in Table 2, the models with AdLIF neurons exhibit significant accuracy improvement than the models employing CUBA-LIF neurons. This highlights that our approach is flexible and works well with various neuron types. This also demonstrates that the FS coding scheme has potential to achieve higher accuracy for data sequences with complex temporal structures if employing advanced architectures and strategies. The comparison results with other state-of-the-art methods is presented in the Appendix.

**Table 6:** Comparison of FR and FS models with AdLIF neurons.

**AdLIF**	**Loss**	**Acc (%)↑**	***N*_*s*_↓**	***t*_*d*_(50*%*)↓**	***t*_*d*_(90*%*)↓**
SHD	FR	90.18	7.24	**28.4**	**57.8**
		FS	**94.08**	**3.65**	55.0	77.2
NTIDIGITS	FR	91.11	13.19	**64.6**	**122.0**
		FS	**92.25**	**6.19**	88.8	129.4

The results are the average of 5 trials. The bold values represent the best results obtained when comparing the FR and FS models for each dataset.

## 4. Conclusion

In this study, we introduce a novel decision-making scheme based on FS coding for realistic event sequences by encoding FS timings of output neurons, and propose a supervised training framework based on FS timings. In the forward pass, discrete temporal coding is applied to the spike trains in the output layer. In the backpropagation, we propose error assignment from FS times to spikes through a Gaussian window and then leverage a surrogate gradient descent method for spikes to achieve supervised learning. Additional strategies are designed to facilitate training and mitigate the influence of inactive neurons, such as adding empty sequences and using different time constants and thresholds for feature extraction and decision layers.

In the experiments, we test the FS coding scheme on classifying various types of event data with rich temporal structures and make a comprehensive comparison with FR coding. Our results provide insights into the distinct mechanisms underlying FS and FR codings. First, FS coding demonstrates comparable performance with FR coding, but there is a trade-off between accuracy and time delay. A relatively longer temporal latency in the first spike helps encode more information, leading to higher FS accuracy. Furthermore, models based on FS and FR codings demonstrate distinct neuronal behavior on different types of data sequences in terms of firing patterns and sparsity. In particular, FS systems are much more energy efficient than FR systems for non-repetitive audio sequences with highly complex temporal structures. In contrast, for visual data sequences with temporal repetition and spatial information, the behavior of FS and FR models is more aligned. The FS systems tend to exhibit longer response time compared to FR systems. Future research could focus on exploring strategies to reduce the temporal delay of the first spike.

## Data availability statement

The original contributions presented in the study are included in the article, further inquiries can be directed to the corresponding authors.

## Author contributions

SL: Conceptualization, Data curation, Formal analysis, Methodology, Software, Validation, Writing—original draft. VL: Supervision, Writing—review and editing. PD: Project administration, Supervision, Writing—review and editing.

## Appendix

For the experiments in Section 3.6, we replaced the CUBA-LIF neurons in hidden layers with AdLIF neurons (Bittar and Garner, 2022) and trained with the FS loss. This adjustment introduces parameters related to adaptation, including adaptation time constant τ_*w*_, and recovery variables *a* and *b*. According to Bittar and Garner (2022), τ_*w*_ is initialized with 100 ms, and *a* and *b* are constrained in the range of *a*∈[−1, 1], *b*∈[0, 2]. However, in our models, we set *a*∈[0, 1] and *b*∈[0, 1]. We observed that this setting leads to more stable training and improved performance. During training, these parameters are trainable along with τ_*m*_.

In particular, for the SHD task, the architecture is 700-FC256-FC256-20, τ_1_ = 5*ms*, τ_2_ = 12τ_1_, θ_1_ = 0.5, θ_2_ = 10. For the NTIDIGITS task, the architecture is 64-FC256-FC256-11, τ_1_ = 5*ms*, τ_2_ = 8τ_1_, θ_1_ = 0.5, θ_2_ = 10. Other training parameters keep the same with the models using CUBA-LIF neurons. In addition, batch normalization (BN) and dropout (DP) are used in each hidden layer. Specifically, a single layer comprises linear operations + BN + neuron + DP. The dropout rate is set to 0.25.

Table A1 presents comparison results of models utilizing CUBA-LIF and AdLIF neurons and the results of other state-of-the-art methods on DVSGesture/SHD/NTIDIGITS datasets. We can see that the performance of models with CUBA-LIF neurons is generally worse, particularly in the case of DVSGesture, indicating that FS coding is not suited for sequences involving temporal repetition. In contrast, models with AdLIF neurons exhibit significant accuracy improvement, achieving comparable performance with state-of-the-art methods, especially for SHD dataset.

**Table A1:** Result comparison of FS models with other methods.

	**Method**	**Acc (%)**
DVSGesture	Hetero. RSNN (Perez-Nieves et al., 2021)	82.9
		SLAYER (Shrestha and Orchard, 2018)	93.64 ± 0.49
		DECOLLE (Kaiser et al., 2020)	95.54
		SLAYER + SpikeMax (Shrestha et al., 2022)	95.83 ± 0.48
		PLIF (Fang et al., 2021) (STBP)	97.57
		STSC-SNN (Yu et al., 2022)	**98.96**
		Ours (CUBA-LIF)	92.8
SHD	Hetero. RSNN (Perez-Nieves et al., 2021)	82.7 ± 0.8
		RSNN with data aug. + noise (Cramer et al., 2022)	83.2 ± 1.3
		Adaptive SRNN (Yin et al., 2021)	90.4
		TA-SNN (Yao et al., 2021)	91.08
		STSC-SNN (Yu et al., 2022)	92.36
		RadLIF (Bittar and Garner, 2022)	94.62
		SNN with learned delays (Hammouamri et al., 2023)	**95.07** **±** **0.24**
		Ours (CUBA-LIF)	87.6
		Ours (AdLIF)	94.08
NTIDIGITS	GRU-RNN (Anumula et al., 2018)	90.9
		Phased-LSTM (Anumula et al., 2018)	91.25
		ST-RSBP (Zhang and Li, 2019)	93.63 ± 0.27
		SLAYER + spike-rate (Shrestha et al., 2022)	**94.19** **±** **0.18**
		SLAYER + SpikeMax (Shrestha et al., 2022)	93.21 ± 0.32
		Ours (CUBA-LIF)	89.3
		Ours (AdLIF)	92.25

The bold values represent the best results for each dataset.
